# An insight on psychiatric insight

**DOI:** 10.1192/j.eurpsy.2021.1270

**Published:** 2021-08-13

**Authors:** T. Coelho Rocha, J. Cunha, S. Torres, A. Lopes

**Affiliations:** Psychiatry And Mental Health Department, Centro Hospitalar Barreiro-Montijo, Barreiro, Portugal

**Keywords:** psychiatric insight, metacognition, phenomenology, awareness of illness

## Abstract

**Introduction:**

Insight is a complex and multidimensional phenomenon. Metacognition, awareness of illness or anosognosia are some of the terms used to designate this feature of the mental state exam.

**Objectives:**

To attempt to explore the evolution of the concept of insight as a psychiatric symptom over the years and to bring up some up-to-date features on this theme.

**Methods:**

Literature review, using the most relevant papers, with the keywords “psychiatric insight”, “awareness of illness”, “metacognition” and “phenomenology”.

**Results:**

The term ‘insight’ has been described since 1896 when Kraepelin had noticed that patients with dementia praecox were unaware of their condition. Nowadays, it is recognized in several psychiatric disorders, with different meanings in each one. Overall, insight in psychiatry involves an attempt to see one’s thinking and behaviour ‘objectively’ and comparing it to some representation of mental health. Impaired insight has been linked to poor treatment compliance and outcomes, overall symptom severity, higher relapse, lower self-esteem, and impaired psychosocial functioning. White matter and connectivity problems may be related to poorer insight, as well as impaired frontal lobe functioning. In psychotic disorders, lack of insight is a primary symptom with poorer outcomes. Regarding affective disorders, the lower the mood the better the insight. Neuroimaging has been correlating insight with the inferior frontal gyrus, anterior insula, inferior parietal lobule, and ventromedial prefrontal cortex. In everyday practice, there are scales used to assess insight.

**Conclusions:**

Inferences about patients’ insight are important to evaluate severity of illness, suicidal risk, compliance, and response to treatment.

